# Impacts of key environmental variables on suitable cultivation and flavonoid accumulation in *Pueraria montana* var. *lobata* under climate change in China

**DOI:** 10.1371/journal.pone.0339508

**Published:** 2026-01-05

**Authors:** Yankun Li, Yuanxin Li, Zhuoyan Chen, Xinyi Yang, Qiuyue Zuo, Mingli Hu, Shimeng Li, Xinyi Teng, Chunsong Cheng, Qiqing Cheng

**Affiliations:** 1 School of Pharmacy, Xianning Medical College, Hubei University of Science and Technology, Xianning, China; 2 Jiangxi Key Laboratory for Sustainable Utilization of Chinese Material Medical Resources, Lushan Botanical Garden, Chinese Academy of Sciences, Jiujiang, China; Anhui University of Chinese Medicine, CHINA

## Abstract

*Pueraria montana* var. *lobata* (*P. lobata*) is both a medicinal herb with significant pharmacological values and a food ingredient that can replace grains, but it still faces challenges in quality consistency and suitable cultivation. This study aims to systematically analyze its potential suitable habitats across China and evaluate the effect of environment on its growth and quality. By integrating distribution data from 926 sample points and 33 environmental variables, MaxEnt model and ArcGIS software were employed to predict the potential suitable habitats of *P. lobata*, and investigate distribution change at the provincial level. Chemical and correlation analysis were used to determine the total flavonoid content and explore the relationship with environmental variables. Key influencing variables were Bio12 (annual precipitation, 35.4%), Bio14 (driest month precipitation, 24%), and Bio06 (coldest month minimum temperature, 18.2%). *P. lobata* from Hubei and Jiangxi provinces exhibited higher flavonoid content than that in other high-suitable provinces, which showed a strong positive correlation with latitude and a significant negative correlation with January mean temperature. Under future climate scenarios, the suitable habitats of *P. lobata* showed northward expansion due to global warming. These findings offer a theoretical foundation for sustainable development and high-quality demand under changing climatic conditions.

## Introduction

*Pueraria montana* var. *lobata* (Willd.) Maesen & S. M. Almeida ex Sanjappa & Predeep, commonly known as *Pueraria lobata* and kudzu, is a perennial woody vine belonging to the Fabaceae family. Its root, known as “Gegen” in traditional Chinese medicine (TCM), has been used therapeutically for centuries and continues to hold significant potential for both culinary and medicinal applications [1]. The pharmacological mechanisms and chemical composition of *P. lobata* have been well documented [2,3]. Modern pharmacological studies have confirmed its bioactive properties, including anticancer [4], anti-inflammatory [5], alcohol-metabolizing [6], antihypertensive [7], hepatoprotective [8,9], hypoglycemic [10]**,** and other therapeutic effects. *P. lobata* produces diverse secondary metabolites, including flavonoids, isoflavonoids, triterpenoids, alkaloids, polysaccharides and coumarins [11]. Among them, flavonoids are the most pharmacologically significant and serve as primary chemical markers for quality assessment in pharmacopoeial standards [12]. In other medicinal plants such as *Ginkgo biloba*, flavonoids have also been shown to exhibit vasodilatory and lipid-regulating effects [13].

However, the sustainable utilization of *P. lobata* faces a crucial dual challenge. While demand for high quality and flavonoid-rich material is growing, cultivated *P. lobata* exhibits heterogeneous quality and often fails to meet pharmacopoeial standards. In contrast, wild resources that satisfy quality requirements are underutilized due to difficulties in harvesting and processing [14]. Although China is rich in *P. lobata* varieties and resources and serve as the global distribution center [15], it is also a highly invasive species in many regions such as the southeastern United States [16,17], where its rapid growth threatens native ecosystems and causes economic losses [18]. The contradiction between its medicinal value and ecological risks underscores the urgent need for a balanced strategy, which can both facilitate controlled cultivation for health benefits and prevent environmental harm.

Species distribution modeling (SDM) offers a powerful tool to address this challenge by predicting suitable habitat distributions based on environmental variables, including climate, soil composition, and topography [19]. Under the background of global warming, the geographical distribution of species is influenced to varying extents, resulting in habitat expansion, contraction, and even migration [20,21]. Among the numerous variables influencing species distribution patterns, climate is one of the most significant determinants. Understanding the potential future distributional changes of species in response to climate variations can inform conservation strategies, enhance species research, and facilitate their sustainable development and utilization [22].

Currently, researchers frequently predict potential suitability zones based on environmental variables, employing widely used models such as Diva Geographic Information System (DIVA-GIS), Climate Extrapolation (CLIMEX), Bioclimatic Envelope Model (BIOCLIM), Genetic Algorithm for Rule-set Production (GARP), and Maximum Entropy (MaxEnt) [23]. Among them, MaxEnt model is particularly advantageous in data-scarce scenarios, renowned for its high predictive accuracy and operational stability [24]. Its applications span diverse studies, including identifying optimal cultivation areas for *Pogostemon cablin* in Guangdong and Hainan provinces [25], predicting distribution decline of *Arisaema heterophyllum* under the future climate scenarios for conservation planning [26], modeling climate change impacts on *Ferulago* and *Thymus* species, and evaluating environmental variables affecting distribution and medicinal compound accumulation in *Schisandra chinensis* [27–29]. These studies demonstrate the versatility and reliability of the MaxEnt model for modeling medicinal and economic plants, assessing climate change impact on species distributions, and evaluating the potential invasive spread. Given these demonstrated capabilities, we selected the MaxEnt model to investigate the future suitable habitats of *P. lobata*.

Considering *P. lobata* possesses both high economic value and significant ecological risk, a critical research gap remains in reconciling these conflicting aspects. At present, there is an urgent need for a comprehensive strategy that can integrate habitat suitability with the chemical quality of plants to clearly guide sustainable cultivation, while also mapping and mitigating the invasive risks under climate change. To fill this gap, our study employs the MaxEnt model and chemical composition analysis of representative production site samples, to identify areas highly suitable for cultivation that maximize flavonoid accumulation, while mitigating potential ecological risks associated with climate-driven expansion. We aim to identify environments most conducive to the accumulation of its active components, thereby guiding controlled, high-quality medicinal cultivation that meets pharmacopoeial standards and reducing unsustainable harvesting of wild resources. Simultaneously, we seek to predict its potential ecological distribution range. This will provide a basis for selecting cultivation sites that avoid ecologically sensitive areas to support ecological conservation efforts. Ultimately, this integrated approach is designed to enhance the overall quality of the medicinal material and offer scientific support for its conservation and sustainable development.

## Results

### Current distribution patterns of *P. lobata* in China

**Fig 1:**
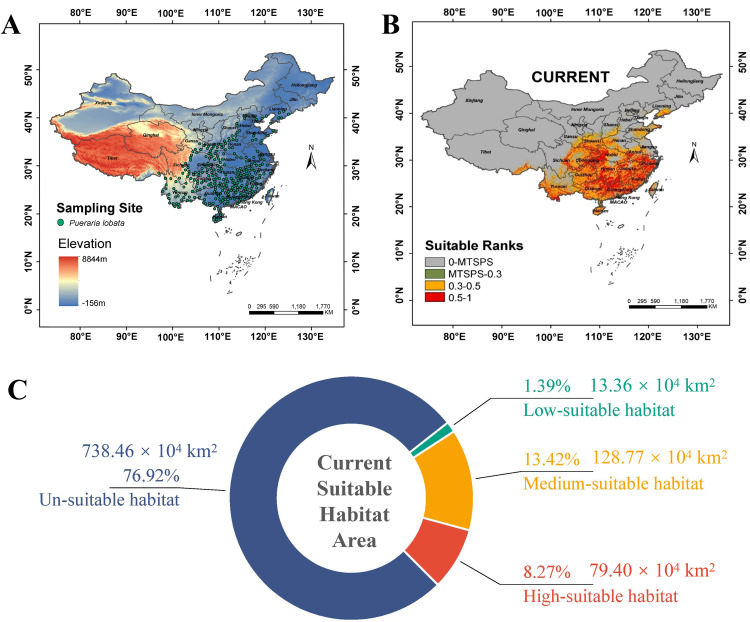
Distribution of *P. lobata* suitable habitats under the current period. **(A)** Geographical distribution of *P. lobata* occurrence records. **(B)** Predicted habitat suitability of *P. lobata* based on the MaxEnt model. Gray areas indicate unsuitable habitats (HSI < 0.2), green areas indicate low-suitable habitats (HSI 0.2–0.4), yellow areas indicate medium-suitable habitats (HSI 0.4–0.6), and red areas indicate high-suitable habitats (HSI > 0.6). **(C)** The area proportions of *P. lobata* under different habitat classifications.

We systematically collected 926 georeferenced occurrence records of *P*. *lobata* from two authoritative databases: the Chinese Virtual Herbarium (CVH; http://www.cvh.ac.cn) and the National Specimen Information Infrastructure (NSII; http://www.nsii.org.cn) (S1 Table). Following standard data screening protocol of our previous study [30], we removed duplicate records and specimens with incomplete geolocation data, yielding 863 validated records (S2 Table), and excluded overlapping points using a 20 km buffer radius to reduce sampling bias [31], resulting in 298 spatially independent occurrences for final analysis (S3 Table and Fig 1A). According to the *Flora of China*, *P. lobata* exhibits a broad distribution range across China except the arid northwestern regions (Xinjiang, Qinghai) and the Qinghai-Tibet Plateau, which is typically found in various forest types, ranging from open woodlands to closed-canopy forests. Its native range extends throughout Southeast Asia to northern Australia and has also invaded the southeastern United States [17]. Based on these occurrences, the MaxEnt model predicted the current habitat suitability of *P. lobata* (Fig 1B). The gray areas represented unsuitable habitat for *P. lobata*, while the green, yellow, and red areas corresponded to low-, medium-, and high- suitable habitats, respectively. The primary distribution range of *P. lobata* spanned from 20°N to 40°N latitude and 100°E to 125°E longitude, covering areas classified as both medium- and high-suitable habitats. Quantitative analysis indicated that the combined area of medium- and high-suitable habitats was approximately 2.08 × 10^6^ km^2^ (21.68% of China’s land area). High-suitable habitat (HSI > 0.6) accounted for 0.79 × 10^6^ km^2^ (8.27% of China’s land area), medium-suitable habitat (HSI 0.4–0.6) for 1.29 × 10^6^ km^2^ (13.42% of China’s land area), and low-suitable habitat (HSI 0.2–0.4) for 1.3 × 10^5^ km^2^ (1.39% of China’s land area) (Fig 1C and Table 1).

**Table 1:** Statistical analysis of suitable areas of *P. lobata* in different periods.

Period		Un-suitable habitat		Low-suitable habitat		Medium-suitable habitat		High-suitable habitat	
		Area (*10^4^ km^2^)	Percentage (%)	Area (*10^4^ km^2^)	Percentage (%)	Area (*10^4^ km^2^)	Percent-age (%)	Area (*10^4^ km^2^)	Percent-age (%)
LGM		916.60	95.48	3.30	0.34	38.20	3.98	1.90	0.20
MH		878.92	91.55	5.82	0.61	74.63	7.78	0.62	0.06
Current		738.46	76.92	13.36	1.39	128.77	13.42	79.40	8.27
2050s	SSP126	727.32	75.76	15.12	1.58	111.84	11.65	105.72	11.01
	SSP585	718.30	74.82	16.40	1.71	121.59	12.67	103.71	10.80
2090s	SSP126	722.28	75.24	15.38	1.60	119.71	12.47	102.63	10.69
	SSP585	690.85	71.96	22.26	2.32	137.01	14.27	109.88	11.45

Note: Area percentages are the ratio of the land surface area of the country occupied by each suitable area in each period.

At present, the distribution of the total suitable area for *P. lobata* was relatively concentrated, predominantly occurring south of the Qinling-Huaihe Line, with the highest concentration in southern and southwestern China. The distribution of the species became increasingly sparse further north, which closely aligned with its natural range as recorded in the *Flora of China*. The high-suitable habitat was primarily concentrated in five provinces, including Guangxi, Hunan, Guangdong, Hubei, and Jiangxi. The medium-suitable habitat was mainly concentrated in four provinces: Anhui, Jiangsu, Zhejiang, and Fujian. The low-suitable habitat and unsuitable habitat were primarily located in the northwest and northeast regions of China. These findings provided a comprehensive understanding for ecological preferences of *P. lobata* and highlighted its concentrated distribution across climatically favorable regions in southern China.

### Environmental variable analysis and selection for *P. lobata* distribution modeling

**Fig 2:**
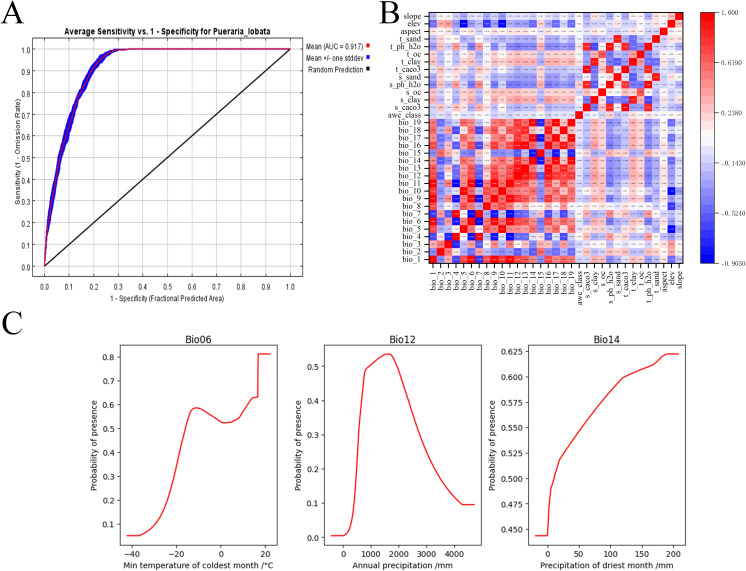
The MaxEnt model prediction results of *P. lobata* and the environmental variables influencing its growth. **(A)** ROC curve of the MaxEnt model; **(B)** Correlations of environmental variables; **(C)** Response curves of key influencing variables.

The MaxEnt model achieved an average training AUC of 0.917 across 10 replicates (Fig 2A), indicating excellent predictive accuracy [32]. This high AUC value confirms the strong predictive capability of model and reliability in simulating the species distribution patterns. The model incorporated 17 environmental variables, with their relative contributions quantified through the Jackknife test (Table 2). Bio12 (annual precipitation) exhibited the highest contribution rate of 35.4%, followed by Bio14 (precipitation of driest month) with a contribution rate of 24%, the Bio06 (min temperature of coldest month) contributed 18.2%. Other contributing variables included slope (slope), elev (elevation), aspect (aspect), Bio03 (isothermality), s_clay (substrate-soil clay content), Bio07 (temperature annual range), Bio02 (mean diurnal range), s_caco3 (topsoil calcium carbonate), and Bio10 (mean temperature of warmest quarter), which contributed 6.2%, 3.9%, 1.8%, 1.8%, 1.6%, 1.4%, 1.3%, 1.2%, 1.1%, and 1.1%, respectively. The contribution rates of the remaining environmental variables were all below 1%. Among these, the permutation importance (%) for Bio12, Bio06, and elevation were comparatively higher, at 40.2%, 13.2%, and 10.7%, respectively. This indicated that the model has a highly significant dependence on these three variables (Fig 2B). The response curve illustrated the range and optimal values of key environmental variables that influenced the distribution of *P. lobata*: annual precipitation ranged from 948.86 mm to 1938.72 mm, precipitation in the driest month varied between 10.58 mm and 123.30 mm, and temperature variation in the coldest month fluctuated between −15.95°C and 12.00°C (Fig 2C).

**Table 2:** Percent contributions and permutation importance (%) of the dominant environmental variables in the MaxEnt model.

Variable	Description	Percent contribution (%)	Permutation importance (%)
Bio12	Annual precipitation	35.4	40.2
Bio14	Precipitation of driest month	24	1.7
Bio06	Min temperature of coldest month	18.2	13.2
slope	slope	6.2	8.6
elev	Elevation	3.9	10.7
aspect	Aspect	1.8	3.0
Bio03	Isothermality (Bio2/Bio7) (* 100)	1.8	1.8
S_clay	Substrate-soil clay content	1.6	1.6
Bio07	Temperature annual range (bio5-bio6)	1.4	2.9
Bio02	Mean diurnal range (mean of monthly (max temp – min temp)	1.3	2.3
s_caco3	Topsoil calcium carbonate	1.2	2.7
Bio10	Mean temperature of warmest quarter	1.1	3.5
t_sand	Sand content	0.8	3.2
t_oc	Topsoil organic carbon	0.5	1.5
awc_class	Soil available water content	0.4	0.4
s_oc	Substrate-soil organic carbon	0.2	0.5
Bio8	Mean temperature of wettest quarter	0.2	2.1

The range of environmental variables associated with an occurrence probability greater than 0.5 was commonly regarded as the threshold for identifying habitats suitable for the species. The probability of occurrence increased as the values of each environmental variable approached the optimal range, but declined when these variables exceeded their respective optimal thresholds. Overall, this analysis confirmed that temperature and precipitation parameters (particularly Bio12, Bio14 and Bio06) serve as the primary environmental determinants governing the distribution of *P. lobata* across the study region.

### Geographic distribution changes of *P. lobata*

Compared with the current distribution, under future climate change scenarios, the medium suitable habitat experienced a significant contraction under the SSP126 scenario, exhibiting an overall decreasing trend. Here, SSP126 and SSP585 correspond to low- and high-greenhouse-gas emission pathways, providing the projected climate context for future habitat suitability. The medium suitable habitat in Hunan, Hubei, and Yunnan provinces underwent a substantial reduction during this period. Both the high and total suitable areas for *P. lobata* have generally expanded. This expansion indicated that, under future climate change scenarios, the medium suitable habitat area could gradually transition into a high-suitable habitat area. In the southern region, a clear trend of increasing total suitable habitat area was observed in Guizhou and Hainan provinces during the future SSP585 period. In the northern region, Liaoning province has shown an upward trend in its suitable habitat area, and the future expansion of *P. lobata* was expected to occur in the suitable growth environments of the northeastern region. Global warming might drive the migration of plant species to higher elevations and latitudes. These patterns emphasize the need to monitor potential invasive spread in newly suitable northeastern habitats, while maintaining conservation efforts in traditional southern core area.

### Historical and current distribution pattern of *P. lobata*

**Fig 3:**
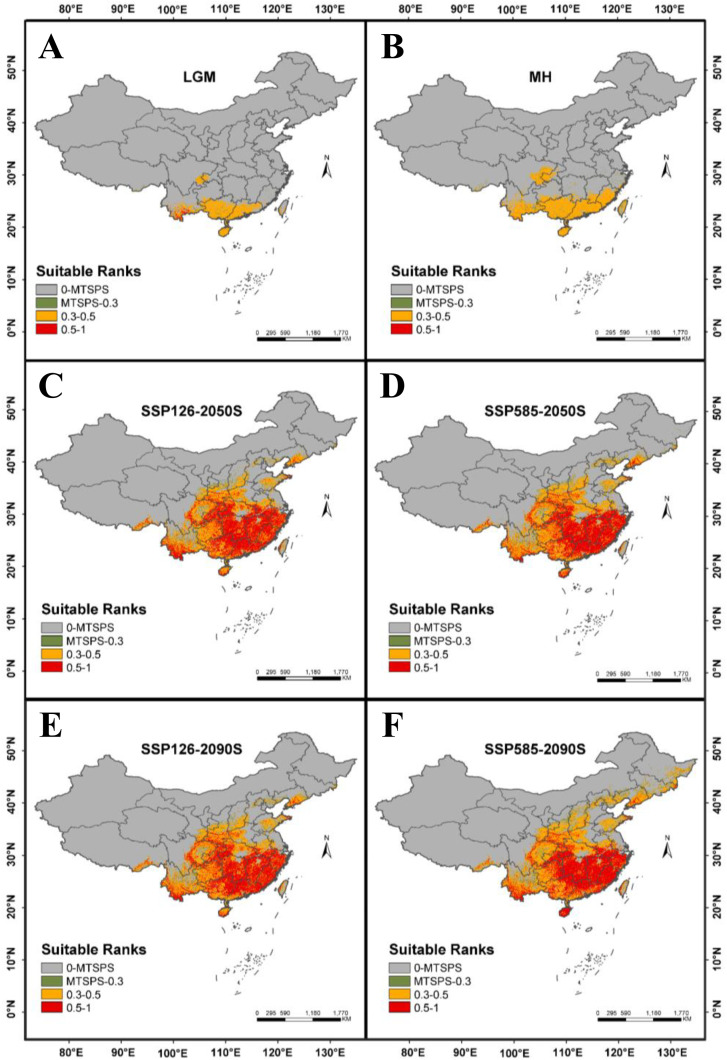
Distribution of suitable habitats for *P. lobata* under different climate scenarios. **(A)**: Last Glacial Maximum (LGM); **(B)**: Mid-Holocene (MH); **(C)**: Average for 2041-2060 (2050s), SSP126; **(D)**: Average for 2041-2060 (2050s), SSP585; **(E)**: Average for 2081-2100 (2090s), SSP126; **(F)**: Average for 2081-2100 (2090s), SSP585.

We selected six distinct time periods to analyze and predict the potential distribution patterns of *P. lobata* across China. From the Last Glacial Maximum (LGM) to the Mid-Holocene (MH) period, the suitable growing area for *P. lobata* exhibited a pronounced southward shift, particularly towards South China. And new suitable regions emerged, primarily concentrated in Fujian and Jiangxi provinces. The LGM period recorded the smallest suitable area, and the sum of high-suitable and medium-suitable area (total suitable area) was only 40.10 × 10^4^ km^2^ (in Fig 3A). In contrast, during the MH period, the total suitable area expanded significantly to 75.25 × 10^4^ km^2^, representing an increase of 35.15 × 10^4^ km² (in Fig 3B). This growth was consistent across low-, medium-, and high-suitable habitat. From the MH period to present, we observed progressive northward expansion of suitable habitats, suggesting that the constrained distributions during LGM and MH periods likely resulted from colder climatic conditions during the LGM [33]. Under future climate scenarios SSP126 and SSP585, the suitable habitats of *P. lobata* are projected to expand notably during the 2050s and 2090s (Fig 3C–3F).

### Future projections under SSP126 and SSP585 scenarios

**Fig 4:**
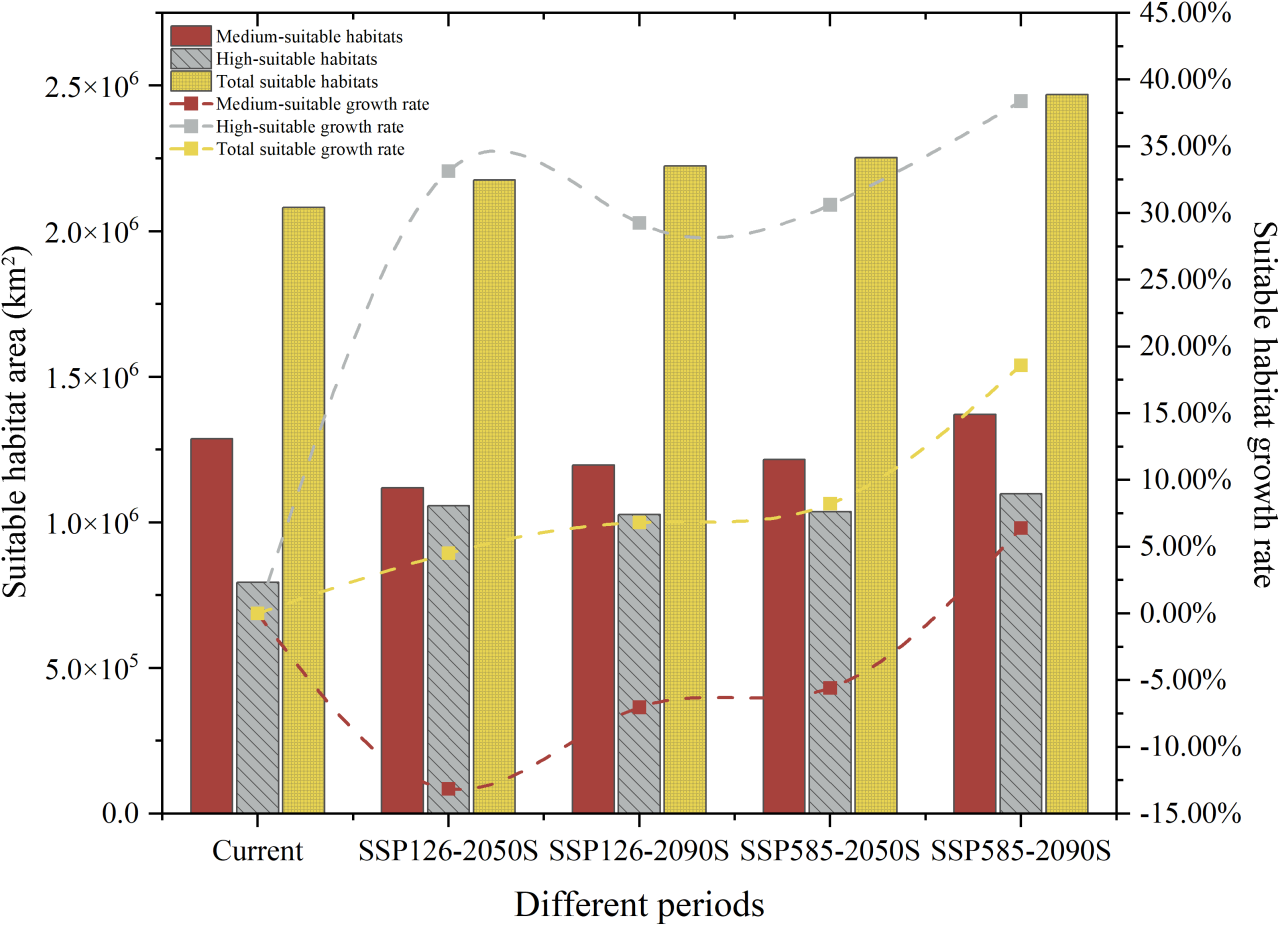
Distribution of suitable areas of *P. lobata* under different climate scenario and different period, along with the growth rates of these areas relative to the current period.

The MaxEnt modeling results revealed significant changes in habitat suitability for *P. lobata* under future climate scenarios SSP126 and SSP585 for two time periods of 2050s and 2090s. All scenarios showed consistent expansion of total suitable areas compared to current conditions, high-suitable habitats demonstrated the most pronounced increases across all projections, and medium-suitable habitats generally decreased, suggesting potential upward transitions to higher suitability classes (Fig 4). Under the SSP126 scenario, the total suitable habitat area for 2041–2060 was projected to be 217.56 × 10^4^ km^2^, representing a 4.51% increase relative to current climate conditions. This projection was primarily driven by an expansion of the highly suitable area, which was estimated to cover 105.72 × 10^4^ km^2^, reflecting an increase of 33.15%. In contrast, the low- and medium-suitable habitat areas were expected to cover 15.12 × 10^4^ km^2^ and 111.84 × 10^4^ km^2^, respectively, with increases of 13.10% and a decrease of 13.36%. The total suitable habitat area for the period 2081–2100 was projected to be 222.35 × 10^4^ km^2^, reflecting a 6.81% increase compared to current climate scenarios. This expansion was primarily attributed to the highly suitable areas, which was estimated to cover 102.63 × 10^4^ km^2^, representing an increase of 29.26%. In contrast, the low and medium suitable areas were expected to cover 15.38 × 10^4^ km^2^ and 119.72 × 10^4^ km^2^, reflecting increases of 15.04% and a decrease of 7.03%, respectively.

Under the SSP585 scenario, the distribution area of *P. lobata* was projected to expand significantly, with the total suitable area reaching 225.29 × 10^4^km^2^ during the period 2041–2060, reflecting an 8.22% increase compared to the current climate scenario. The high-suitable area was expected to cover 103.71 × 10^4^ km^2^, representing a 30.62% increase. In contrast, the low-suitable and medium-suitable areas were projected to cover 16.40 × 10^4^ km^2^ and 121.59 × 10^4^ km^2^, reflecting increases of 22.75% and a decrease of 5.58%, respectively. The total suitable habitat area for *P. lobata* during the 2081–2100 period was projected to reach 246.89 × 10^4^ km^2^, indicating an 18.60% increase compared to the current climate scenario. The high-suitable area was expected to span 109.88 × 10^4^ km^2^, reflecting a 38.38% increase. In contrast, the low-suitable and medium-suitable areas were projected to occupy 22.26 × 10^4^ km^2^ and 137.01 × 10^4^ km^2^, respectively, with increases of 66.62% and 6.39%.

We summarized the suitable distribution areas of *P. lobata* for each time period, along with the growth rates of these areas relative to the current period (Fig 4). Additionally, we analyzed the changes in the suitable areas of *P. lobata* across different provinces in China, finding that its primary distribution was concentrated in central and southern China, with a notable future shift northward, particularly towards the northeastern region of China. Suitable habitat areas for the species have now been identified across the three northeastern provinces. At this point, the total suitable habitat area for *P. lobata* has reached its maximum extent [34]. Overall, the total suitable habitat area has shown a consistent increase, with the high-suitable area continuing to expand. This trend suggests that future climatic conditions are likely to facilitate the growth of *P. lobata*.

### Provincial distribution patterns of *P. lobata* in China

**Fig 5:**
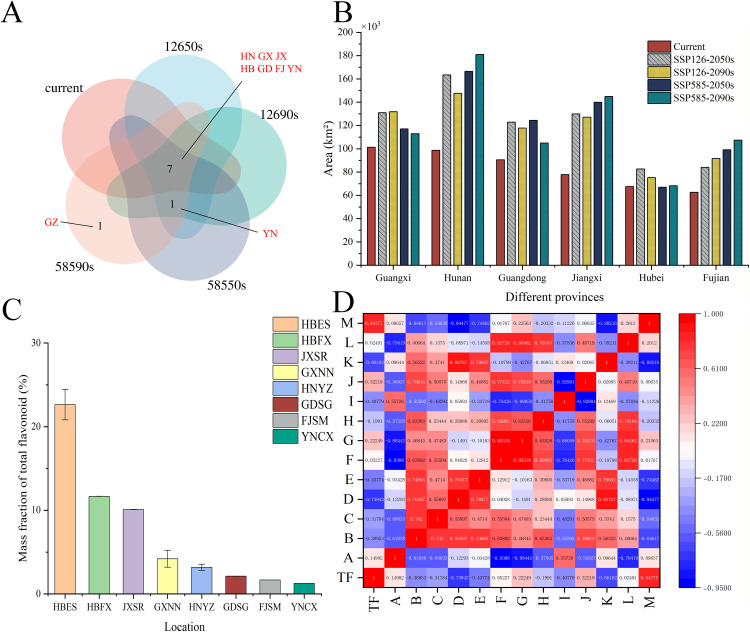
Factors influencing total flavonoid content in *P. lobata*, high-suitable provinces, and distribution area. **(A)** Suitable habitats of *P. lobata* in each province during different time periods. **(B)** Venn diagram of provinces with a highly suitable area for *P. lobata* exceeding 60,000 km² across different periods. (HN: Hunan, GX: Guangxi, JX: Jiangxi, HB: Hubei, GD: Guangdong, FJ: Fujian, YN: Yunnan). **(C)** Comparison of total flavonoid content in the most suitable provinces for *P. lobata* growth. HBES, HBFX, JXSR, GXNN, HNYZ, GDSG, FJSM, and YNCX represented the *P. lobata* samples from Enshi city in Hubei province, Fang-xian in Hubei province, Shangrao city in Jiangxi province, Nanning city in Guangxi zhuang autonomous region, Yongzhou city in Hunan province, Shaoguan city in Guangdong province, Sanming city in Fujian province, and Chuxiong city in Yunnan province respectively. **(D)** Correlation analysis of climatic variables with the total flavonoid content of *P. lobata* from various origins.[TF: Total flavonoids, A: Elevation **(m)**, B: Mean annual temperature (°C), C: Annual cumulated temperature (°C), D: Mean January temperature (°C), E: January minimum temperature (°C), F: Mean July temperature (°C), G: July maximum temperature (°C), H: Annual precipitation (mm), I: Annual sunshine duration **(h)**, J:Mean annual relative humidity (%), K: Frost-free period (days), L: Longitude, M: Latitude].

The provincial distribution pattern of *P. lobata* suitable habitat in China were analyzed and shown (Fig 5A). Our MaxEnt model projections identified 13 provinces with high-suitable habitats exceeding 10,000 km^2^ across five scenarios: Guangxi, Hunan, Guangdong, Jiangxi, Hubei, Fujian, Yunnan, Zhejiang, Sichuan, Guizhou, Chongqing, Anhui, and Shaanxi. Among them, Guangxi exhibited the largest high-suitable habitat area with 101,230 km^2^. Under future climate scenarios, most provinces showed expansions in high-suitable habitats. Specifically, SSP585-2090s projections revealed more than 60% substantial increases in Hunan (83%), Jiangxi (86%), Fujian (72%), and Guizhou (61%) compared to current situation. Some provinces with moderately sized high-suitable habitats (1,000–10,000 km^2^), including Hainan, Liaoning, and Gansu, demonstrated even more dramatic expansions under SSP585-2090s, with increases exceeding 100% (Hainan: 261%; Liaoning: 234%). Remarkably, Heilongjiang and Jilin—currently lacking high-suitable habitats—projected new potential ranges of 2,053 km^2^ and 1,570 km^2^, respectively under SSP585-2090s. These findings highlight a pronounced northward shift in suitable habitats, driven by climate warming, while reinforcing the dominance of southern provinces as core distribution zones.

### Correlation analysis between climatic variables and total flavonoids of *P. lobata* from different origins

Our study systematically evaluated the relationship between habitat suitability and medicinal quality of *P. lobata* across primary production regions of China. We statistically analyzed the high-suitable habitats across five distinct periods (LGM, MH, current, 2050s, and 2090s) and identified seven provinces where the high-suitable habitats area for *P. lobata* consistently exceeded 60,000 km^2^, including Guangxi, Hunan, Guangdong, Jiangxi, Hubei, Fujian, and Yunnan (Fig 5B). These provinces represent the core distribution zones of *P. lobat*a in China. Under future climate scenarios (SSP126-2050s, SSP126-2090s, SSP585-2050s, SSP585-2090s), these provinces maintained their dominance while showing significant expansion, underscoring their enduring suitability for cultivation.

We quantified total flavonoid content in representative samples from eight production sites across the seven high-suitability provinces (HBES, HBFX, JXSR, GXNN, HNYZ, GDSG, FJSM, YNCX). Samples from Enshi (HBES) and Fangxian (HBFX) in Hubei province showed the highest total flavonoid contents (22.63% and 11.67%, respectively), followed by Shangrao (JXSR, 10.10%). Samples from the remaining sites showed lower values (GXNN 4.20%, HNYZ 3.17%, GDSG 2.11%, FJSM 1.67%, YNCX 1.26%) (Fig 5C). Correlation analysis revealed significant associations between flavonoid accumulation and climatic conditions. Latitude was positively correlated with total flavonoid content (r = 0.8478), while mean January temperature was negatively correlated (r = −0.7394) (Fig 5D). These results indicate that, beyond broad habitat suitability, specific microclimatic factors—particularly latitude and winter temperature—are critical determinants of medicinal quality.

## Discussion

### Environmental determinants of *P. lobata* suitable habitat distribution

As climatic variables play a crucial role in shaping species distribution [35], this study presents a comprehensive analysis of the environmental variables governing the distribution and medicinal quality of *P. lobata* in China. Our MaxEnt modeling revealed that precipitation parameters (Bio12, Bio14) and minimum winter temperature (Bio6) collectively explain 77.6% of habitat suitability, consistent with its subtropical origin and physiological requirements, which aligns with the factors affecting the potential suitable planting areas of *Alternanthera philoxeroides* and *Litsea cubeba* [36,37]. The current geographic distribution of *P. lobata* is primarily concentrated in the East China, South China, and Southwest regions, which are characterized by a subtropical monsoon climate. These areas are marked by distinct seasons, with hot and humid summers, mild and dry winters, and abundant precipitation [38,39]. These climatic conditions align with the growth habits of *P. lobata*. Some researchers have suggested that *P. lobata* grows well under conditions where the annual precipitation ranges from 1000 to 1500 mm when studying its potential distribution and environmental threats [40]. The variation in the minimum temperature during the coldest month ranges from −15.95°C to 12.00°C, with the suitable growth area of *P. lobata* in northern regions being significantly smaller than in southern regions. This suggests that *P. lobata* is highly sensitive to frost. *P. lobata* is not suitable for regions with significant temperature fluctuations and low temperatures, as excessively low temperatures hinder the expansion and development of its rhizomes. In summary, the dominant variables discussed above suggest that *P. lobata* thrives in environments characterized by high annual precipitation, a broad range of annual precipitation, and moderate seasonal temperature variations. The impact of environmental variables, such as precipitation and temperature, should be thoroughly considered in future efforts to introduce *P. lobata* cultivation and resource conservation.

### Climate change impacts and range shifts of *P. lobata*

Compared to the current climate conditions, the future high-temperature environment induced by carbon emissions is expected to result in an overall expansion of the suitable habitat for *P. lobata.* Under the low-emission SSP126 scenario, the suitable habitat area for *P. lobata* is projected to exhibit an upward trend in the future, and the distribution of *P. lobata* in the 2090s period shows an increase of 6.81% in the total suitable area compared with the current period, of which the area of highly suitable area increases by 29%, which indicates that the climatic conditions in this period further expand the highly suitable area compared with the current period. Under the high-emission SSP585 scenario, the suitable habitat area for *P. lobata* is expected to continue exhibiting an upward trend in the future, and the growth rate of the suitable area was more obvious compared with that of the scenario of SSP126, and the area of the total suitable area in the period of 2090s reached the peak, which was an increase of 18.60% compared with the current one. The new area of suitable habitat showed a tendency of spreading from south to north, particularly northward into Heilongjiang and Jilin provinces, which the expansion mirrors patterns were also observed in thermophilic species [41]. In addition to the general effects of increasing temperature and changing precipitation, the northward migration of *P. lobata* is closely tied to its inherent ecological traits. This species is highly adaptable to diverse soil and climatic conditions, which enables it to establish rapidly in newly suitable habitats. Warmer temperatures lengthen the frost-free period, enhancing overwinter survival of roots and rhizomes that would otherwise be limited by harsh winters. Altered precipitation regimes, including increased summer rainfall in some regions, further facilitate seedling establishment and clonal spread. Moreover, *P. lobata* exhibits an exceptionally rapid growth rate and prolific vegetative reproduction, allowing it to quickly exploit extended growing seasons and available resources. Its invasive characteristics, particularly the ability to overtop and suppress native vegetation, further amplify its success once introduced into novel environments. Several studies have demonstrated that not only invasive species but also crops and natural forest communities are experiencing northward or higher-latitude/altitude shifts under climate change. For example, in China, the potential northern planting boundaries of major crops such as winter wheat, spring maize, and double/triple rice have shifted markedly between the periods 1961–1990 and 1991–2020, with northward movements ranging from approximately 20–300 km in regions such as Northeast China and Inner Mongolia [42]. In boreal forest ecosystems, long-term satellite monitoring revealed that from 1985 to 2020, tree cover expanded significantly northward, with an average latitudinal shift of about 0.29° (mean) to 0.43° (median) [43]. Similarly, in the Santa Rosa Mountains of southern California, a comparison of surveys conducted in 1977 and 2006–2007 showed that dominant plant species shifted their elevational ranges upward by an average of 65 m, reflecting expansion toward cooler, higher-altitude habitats [44].

Considering the invasive potential of *P. lobata*, it may lead to a paradoxical scenario—while offering new cultivation opportunities, may also raise ecological concerns. Historical precedents of invasive spread in the southeastern U.S. demonstrate its capacity to outcompete native flora through rapid vegetative growth and dense canopy formation. If similar dynamics were to occur in northeastern China, such expansion could pose risks to native understory vegetation and the potential for genetic introgression with indigenous *Pueraria* species. Our MaxEnt projections indicate increased climatic suitability in parts of northeastern China (e.g., Jilin and Heilongjiang); however, these projections reflect potential climatic suitability rather than confirmed establishment. Actual expansion will depend on factors not captured by our climate-only SDM—such as propagule pressure, land-use and disturbance regimes, and extreme winter minima—which may constrain or facilitate establishment. Therefore, we recommend implementing preventive, model-based early monitoring measures in newly projected suitable areas, integrating systematic on-site surveys, remote sensing screenings, and on-site verifications to identify and address the initial population conditions. This preventive approach helps to strike a balance between the demand for medicinal plant cultivation and ecological protection.

### Medicinal quality determinants of *P. lobata*

Our comprehensive analysis reveals that January low temperature (Bio06) and latitude are pivotal environmental determinants governing both the geographic suitability and medicinal quality of *P. lobata* in China. The strong positive correlation between total flavonoid content and latitude (r = 0.8478), coupled with the negative correlation with January mean temperature (r = −0.7394), suggests a clear latitudinal gradient in medicinal quality. This pattern likely reflects the combined effects of colder winter temperature and greater seasonal variability at higher latitudes, which appear to stimulate flavonoid biosynthesis as part of the plant’s adaptive stress response [45,46].

The superior quality observed in Hubei and Jiangxi provinces can be attributed to their optimal latitudinal position (28–32°N) and characteristic cold winters (mean January temperatures of 2–6°C) [47]. These conditions may trigger several physiological responses, such as cold stress enhanced phenylalanine ammonia-lyase activity by cold stress promoting flavonoid precursor synthesis, prolonged winter dormancy facilitating resource allocation to secondary metabolites, and greater diurnal temperature variation stimulating defensive compound production [48,49].

These findings have significant implications for *P. lobata* cultivation and quality control. The 28–32°N belt emerges as the premium region for both biomass and medicinal-grade production. Future warming may necessitate northward migration of cultivation areas to maintain quality standards. January temperature and latitude can serve as reliable indicators for predicting flavonoid content in new cultivation areas.

### Limitations and future directions

**Fig 6:**
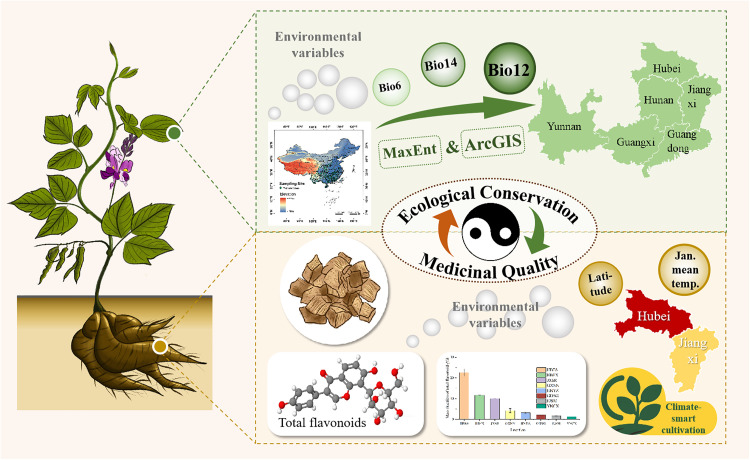
Climate-driven ecological conservation and medicinal quality optimization of *P. lobata* in China.

While our MaxEnt model exhibited strong predictive accuracy (AUC = 0.917), several limitations should be acknowledged to guide future research. Although climatic and edaphic factors accounted for a substantial portion of habitat suitability, our model did not incorporate genetic differentiation data. Given that *P. lobata* exhibits significant differences in flavonoid medicinal components and genetic information [50,51], integrating genomic analyses—such as population-level transcriptomics or single-nucleotide polymorphism (SNP) markers—could enhance regional suitability predictions by identifying locally adapted genotypes. Moreover, we identified strong correlations between latitude, temperature and flavonoid content (Fig 6), further research should focus on several aspects, including molecular mechanisms of cold-induced flavonoid synthesis, genotype-environment interactions across the latitudinal gradient, and development of latitudinally adapted cultivars for climate resilience.

This study bridges critical gaps between biogeographic modeling and phytochemical research, advancing a framework for “climate-smart” cultivation of medicinal plants. By integrating habitat suitability with pharmacological quality metrics, we identify priority regions of Hubei and Jiangxi provinces, where ecological conservation and medicinal quality can be synergistically optimized (Fig 6). Future efforts should expand this approach to other economically important agricultural and medicinal species, incorporating genetic, phenotypic, and climatic data to build effective strategies that balance sustainable cultivation and species quality.

## Materials and methods

### Acquisition and screening of distribution data

Distribution data for *P. lobata* were obtained from the China Digital Herbarium (CVH, http://www.cvh.ac.cn/) and the National Specimen Information Infrastructure (NSII, http://www.nsii.org.cn/), yielding a total of 928 initial records. All specimens were taxonomically identified by professional botanists, and voucher specimen numbers and herbarium information are provided (S1 Table). To ensure data reliability, duplicate entries, records with incomplete or ambiguous locality descriptions, and samples whose geographic descriptions did not match the recorded coordinates were removed. For specimens lacking coordinate data but providing clear locality descriptions, geographic coordinates were determined and verified using the Baidu Coordinate Picker system (https://api.map.baidu.com/lbsapi/getpoint/index.html). After coordinate verification and removal of duplicate or ambiguous records, 863 records with valid locality information were retained (S2 Table).

To ensure that the final dataset represented wild-growing populations, metadata fields, specimen remarks, and locality notes were examined and records clearly associated with managed or cultivated environments (e.g., nurseries, experimental stations, campuses, or botanical gardens) were excluded. To reduce spatial clustering, the 863 valid points were imported into ArcGIS 10.4.1 and a 20-km buffer was applied; within each buffer only one representative point was retained to avoid oversampling of densely collected areas [52]. This procedure resulted in 298 validated wild distribution points (S3 Table). Finally, the species name, longitude, and latitude of the retained records were compiled into a standardized spreadsheet and exported in.CSV format for subsequent ecological niche modelling analyses.

### Acquisition and screening of environmental variables

Nineteen climate variables were obtained from the WorldClim database (http://www.worldclim.org), using current (1970–2000) climate data as the baseline. Additionally, different climate scenarios for LGM, MH, and future (2050s, 2090s) were selected for comparison. Nineteen climate variables were considered under different scenarios. Specifically, future climate data were derived from the Shared Socio-Economic Pathways (SSPs) model, as published by the Sixth Coupled Model Intercomparison Project (CMIP6). Among the SSP scenarios, SSP126 represents a low-emission pathway, while SSP585 corresponds to a high-emission pathway, reflecting the most optimistic and pessimistic projections of future greenhouse gas (GHG) emissions, respectively [53]. Eleven soil variables were obtained from the Harmonized World Soil Database (HWSD, v1.2; FAO/IIASA) with an original resolution of 30 arc-seconds (~1 km). All variables were resampled to a uniform 2.5 arc-minutes (~5 km) resolution to match the climatic layers from WorldClim v2.1.Eleven soil variables were obtained from the World Soil Database (http://www.fao.org/soils-portal/data-hub/en/). Three topographic variables (elevation, slope, and aspect) were derived from the Shuttle Radar Topography Mission (SRTM DEM, CGIAR-CSI v4.1) with an original resolution of 90 m (~3 arc-seconds). Slope and aspect were calculated from the original DEM using ArcGIS software. A total of 33 independent environmental variables were selected for further analysis in this study (Table 3).

**Table 3:** Description of the environmental data.

Variable	Description	Unit	Variable	Description	Unit
Bio1	Annual mean temperature	°C	Bio18	Precipitation of warmest quarter	mm
Bio2	Mean diurnal range (mean of monthly (max temp – min temp))	°C	Bio19	Precipitation of coldest quarter	mm
Bio3	Isothermality (Bio2/Bio7) (* 100)	%	awc_class	Soil available water content	%
Bio4	Temperature seasonality (standard deviation * 100)	%	s_caco3	Topsoil calcium carbonate	%
Bio5	Max temperature of warmest month	°C	s_clay	Substrate-soil clay content	%
Bio6	Min temperature of coldest month	°C	s_oc	Substrate-soil organic carbon	%
Bio7	Temperature annual range (bio5-bio6)	°C	s_ph_h2o	Substrate-soil pH	
Bio8	Mean temperature of wettest quarter	°C	s_sand	Sediment content in the subsoil	%
Bio9	Mean temperature of driest quarter	°C	t_caco3	Topsoil carbonate or lime content	%
Bio10	Mean temperature of warmest quarter	°C	t_clay	Clay content in the upper soil	%
Bio11	Mean temperature of coldest quarter	°C	t_oc	Topsoil organic carbon	%
Bio12	Annual precipitation	mm	t_ph_h2o	Topsoil pH	–
Bio13	Precipitation of wettest month	mm	t_sand	Sand content	%
Bio14	Precipitation of driest month	mm	aspect	Aspect	°
Bio15	Precipitation seasonality (coefficient of variation)	%	elev	Elevation	m
Bio16	Precipitation of wettest quarter	mm	slope	Slope	°
Bio17	Precipitation of driest quarter	mm			

To mitigate potential overfitting of the model resulting from high correlations and multicollinearity among environmental variables, and to ensure prediction accuracy, Spearman’s rank correlation analysis was performed on the aforementioned climatic and soil variables using SPSS 26.0 software. Environmental variables with correlation coefficients (|r| > 0.8) and lower contribution rates were excluded, while all topographic variables were retained. After screening, a total of 17 environmental variables were selected for model construction, including 8 climate variables (Bio2, Bio3, Bio06, Bio07, Bio08, Bio10, Bio12, Bio14), 6 soil variables (awc_class, s_clay, s_oc, t_sand, s_caco3, t_oc), and 3 topographic variables (aspect, elev, slope) [54].

### Construction of the MaxEnt model

The distribution data of *P. lobata* and the relevant environmental variables were imported into MaxEnt (V3.4.3) software to predict the potential suitability areas for *P. lobata*. Modeling settings were as follows: the bootstrap method was used for replicate runs, with 10 replicates; for each replicate, 75% of occurrence points were randomly selected as the training set and the remaining 25% as the test set. We used logistic output format and automatic feature selection. The maximum number of iterations was set to 500 and the convergence threshold to 1 × 10^−5^. For each replicate the model output was saved and the mean prediction across 10 replicates was used as the final suitability map [55]. In this study, the area under the receiver operating characteristic (ROC) curve (AUC) was used to assess the accuracy of the model’s predictions. Additionally, the Jackknife method was employed to evaluate the impact of each environmental variable on the distribution of *P. lobata*, and response curves for the key environmental variables were generated. In addition, the Maximum Test Sensitivity Plus Specificity (MTSPS) Logistic Threshold was applied to classify the suitable distribution area of *P. lobata* into four categories: unsuitable habitat (0 ~ MTSPS), low-suitable area (MTSPS ~ 0.3), medium-suitable area (0.3 ~ 0.5), and high-suitable area (0.5 ~ 1). The areas corresponding to each suitable distribution area were then calculated [56]. We applied the Maximum Test Sensitivity Plus Specificity (MTSPS) Logistic Threshold to classify habitat suitability. MTSPS was chosen because it balances sensitivity and specificity, providing an objective classification. A brief check showed that using the conventional HSI > 0.6 threshold gave similar results, confirming robustness. Model evaluation: For each model run, AUC values were calculated for both training and test datasets to evaluate predictive performance. An AUC value of 0.5 indicates performance no better than random, 0.7–0.9 indicates moderate predictive ability, and values above 0.9 indicate excellent predictive performance. The final AUC values reported were averaged across 10 replicate runs to ensure robustness and reproducibility.

### Habitat suitability analysis of *P. lobata* in different provinces of China

ArcGIS 10.4.1 software was utilized to systematically analyze the distribution of suitable habitats across various provinces in China. Prior to area calculations, all raster layers were reprojected to the WGS 1984 Equal Area Conic projection to ensure accurate area estimation. Following the modeling of species distribution, spatial changes between current and future habitats were quantified using ArcGIS 10.4.1. The predicted results for both current and future habitat conditions were imported into ArcGIS as ASCII files, which were then converted to raster format and reclassified into four categories: Unsuitable habitat, low habitat suitability, medium habitat suitability, and high habitat suitability. To compare the variations in area and distribution between the future and current suitability categories, these were further reclassified into numerical values: 0, 1, 2, 3, and 4. The current habitat suitability layer was then subtracted from the future habitat suitability layer using the “Subtraction” tool within ArcToolbox [57]. Area calculations for both suitable habitats and the subtraction results were conducted using ArcGIS 10.4.1. Subsequently, the Zonal Statistics as Table tool was applied to compute the total area of each suitability category within the provincial boundaries, and all base maps used for spatial analysis were obtained from the Standard Map Service System of the Ministry of Natural Resources of China (http://bzdt.ch.mnr.gov.cn/, Map Approval No.: GS(2019)1822).

### Determination of total flavonoid contents in *P. lobata*

This study references the methodology for the determination of total flavonoid content in *P. lobata*, as reported by Pei et al [58]. Approximately 1.0 g of powdered *P. lobata* sample was accurately weighed and extracted with 60% ethanol solution by ultrasonic treatment for 30 min, followed by filtration. The samples included *P. lobata* from representative production areas in Jiangxi, Hubei, Hunan, Guangdong, Guangxi, Fujian and Yunnan provinces. Puerarin (≥98%) was used as the reference standard. A standard curve was prepared in the range of 0.3125–10 mg·L^-1^, with the regression equation Y = 93.605X + 0.0018 (r^2 ^= 1). Absorbance was measured at 250 nm using 60% ethanol as the blank. Each sample was tested in triplicate, and mean values were used to calculate total flavonoid content.

### Correlation analysis between flavonoid content and climatic variables

Representative samples were collected from eight production sites across seven high-suitable provinces (Hubei Enshi, Hubei Fangxian, Jiangxi Shangrao, Guangxi Nanning, Hunan Yongzhou, Guangdong Shaoguan, Fujian Sanming, Yunnan Chuxiong).Climatic data for the sampling sites were obtained from the National Meteorological Center of China (http://data.cma.cn/). The environmental variables included in the correlation analysis were elevation (m), mean annual temperature (°C), annual accumulated temperature (°C), mean January temperature (°C), January minimum temperature (°C), mean July temperature (°C), July maximum temperature (°C), annual precipitation (mm), annual sunshine duration (h), mean annual relative humidity (%), and frost-free period (days). Each sampling site was paired with its corresponding climatic parameters, and Pearson correlation coefficients between total flavonoid content and each environmental variable were calculated using SPSS 26.0. Statistical significance was set at P < 0.05.

## Conclusions

This study systematically evaluated the impacts of key environmental variables on the suitable cultivation and flavonoid accumulation of *Pueraria montana* var. *lobata* (*P. lobata*) under climate change scenarios in China. Using MaxEnt modeling and ArcGIS analysis, we identified annual precipitation (Bio12, 35.4%), driest month precipitation (Bio14, 24%), and coldest month minimum temperature (Bio06, 18.2%) as the dominant factors shaping its distribution. The model prediction results revealed a northward expansion of suitable habitats under future climate scenarios (SSP126 and SSP585). Notably, high-suitable regions in Hubei and Jiangxi provinces exhibited superior flavonoid content (10.32–22.63%), strongly correlated with higher latitude (r = 0.8478) and lower January temperatures (r = −0.7394). Our study revealed the dual role of climate change in expanding planting potential and improving species quality. These findings not only highlight the importance of combining habitat suitability with phytochemical quality assessment, but also emphasize the necessity of optimizing climate resources and environmental conditions for the sustainable cultivation and utilization of *P. lobata.* Therefore, it provides a scientific basis for carrying out climate-intelligent cultivation under the trend of climate change to support the sustainable development of *P. lobata*.

## Supporting information

S1 Table Distribution information of all *Pueraria montana* var. *lobata* points collected in this study (926 samples). (XLSX)

S2 Table Distribution information of *Pueraria montana* var. *lobata* excluding the duplicate points and unknown information points (863 samples). (XLSX)

S3 Table Distribution records of *Pueraria montana* var. *lobata* after removing overlapping points within 20 km (298 samples). (XLSX)

S4 Table Environmental variables for samples from high-suitable provinces. (XLSX)
